# Thymoquinone, a Dietary Bioactive Compound, Exerts Anti-Inflammatory Effects in Colitis by Stimulating Expression of the Colonic Epithelial PPAR-γ Transcription Factor

**DOI:** 10.3390/nu13041343

**Published:** 2021-04-17

**Authors:** Balaji Venkataraman, Saeeda Almarzooqi, Vishnu Raj, Abdullah T. Alhassani, Ahmad S. Alhassani, Khadijah J. Ahmed, Veedamali S. Subramanian, Shreesh K. Ojha, Samir Attoub, Thomas E. Adrian, Sandeep B. Subramanya

**Affiliations:** 1 Department of Physiology, College of Medicine and Health Sciences, United Arab Emirates University, Al Ain PO Box 17666, United Arab Emirates; balajiv@uaeu.ac.ae (B.V.); rajvishnu@uaeu.ac.ae (V.R.); 2 Zayed Bin Sultan Center for Health Sciences, College of Medicine and Health Sciences, United Arab Emirates University, Al Ain PO Box 17666, United Arab Emirates; 3 Department of Pathology, College of Medicine and Health Sciences, United Arab Emirates University, Al Ain PO Box 17666, United Arab Emirates; saeeda.almarzooqi@uaeu.ac.ae; 4 College of Medicine and Health Sciences, United Arab Emirates University, Al Ain PO Box 17666, United Arab Emirates; abdullah.t1@outlook.com (A.T.A.); 201319259@uaeu.ac.ae (A.S.A.); ahmed-ad879@hotmail.com (K.J.A.); 5 Department of Medicine, University of California, Irvine, CA 92697, USA; vsubrama@uci.edu; 6 Department of Pharmacology and Therapeutics, College of Medicine and Health Sciences, United Arab Emirates University, Al Ain PO Box 17666, United Arab Emirates; shreeshojha@uaeu.ac.ae (S.K.O.); samir.attoub@uaeu.ac.ae (S.A.); 7 Department of Basic Medical Sciences, Mohamed Bin Rashid University of Medicine and Health Sciences, Dubai PO Box 505055, United Arab Emirates; Thomas.Adrian@mbru.ac.ae

**Keywords:** Thymoquinone, DSS colitis, PPAR-γ expression, PPAR-γ promoter, MAPK and colitis

## Abstract

Inflammatory bowel diseases (IBD) are chronic inflammatory disorders with increasing incidence and prevalence worldwide. Here, we investigated thymoquinone (TQ), a naturally occurring phytochemical present in *Nigella sativa*, for anti-inflammatory effects in colonic inflammation. To address this, we used in vivo (mice) and in vitro (HT-29 cells) models in this investigation. Our results showed that TQ treatment significantly reduced the disease activity index (DAI), myeloperoxidase (MPO) activity, and protected colon microscopic architecture. In addition, TQ also reduced the expression of proinflammatory cytokines and mediators at both the mRNA and protein levels. Further, TQ decreased phosphorylation of the activated mitogen-activated protein kinase (MAPK) signaling pathway and nuclear factor kappa B (NF-κB) proteins and enhanced colon epithelial PPAR-γ transcription factor expression. TQ significantly decreased proinflammatory chemokines (CXCL-1 and IL-8), and mediator (COX-2) mRNA expression in HT-29 cells treated with TNF-α. TQ also increased HT-29 PPAR-γ mRNA, PPAR-γ protein expression, and PPAR-γ promoter activity. These results indicate that TQ inhibits MAPK and NF-κB signaling pathways and transcriptionally regulates PPAR-γ expression to induce potent anti-inflammatory activity in vivo and in vitro models of colon inflammation.

## 1. Introduction

Inflammatory bowel disease (IBD) covers a broad category of debilitating disorders that comprises Crohn’s disease (CD), which associates any segment of the gastrointestinal tract, and ulcerative colitis (UC), which involves only the colon (large intestine) and rectum [1]. To date, the exact cause of IBD and its pathogenesis is not entirely understood but appears to be multifactorial [2]. IBD is suggested to result from an uncontrolled immune response in genetically prone individuals, with environmental factors playing a triggering role [3]. IBD also predisposes to the development of colorectal cancer [4]. Current drug therapies available for IBD treatment include sulfasalazine, corticosteroids, as well as immunosuppressive and biological agents. These drugs are targeted to mitigate the observed aberrant immune response that leads to inflammation [4]. However, adverse effects of these drugs over prolonged treatment periods, and the high relapse rate of IBD, limit their use in the long-term. It is estimated that up to 40% of IBD patients seek unconventional treatments [5].

Recent reports show low expression of PPAR-γ in the colon of UC patients, suggesting an inverse correlation between PPAR-γ and UC progression [6,7]. PPAR-γ is a known modulator of cytokine/chemokine production [6,7], making it a useful therapeutic target for IBD [8]. Some phytochemical ligands that modulate the PPAR-γ pathway have been shown experimentally to limit colonic inflammation. Thymoquinone (TQ) is one such phytochemical that acts as a PPAR-γ agonist, based on experimental and *in silico* docking studies [9,10]. TQ (2-isopropyl-5-methyl-1,4-benzoquinone) is the principal bioactive molecule (a member of the quinone–monoterpene family) present in volatile oil of *Nigella sativa* L. (*Ranunculaceae* family) seeds, known as black seed or black cumin, and popularly used in Mediterranean and Middle Eastern countries for its health benefits [11]. TQ has broad nutraceutical properties, including antioxidant, anti-inflammatory, anticancer, antimicrobial, and anti-diabetic effects [12]. TQ-rich *Nigella sativa* oil increases gastric mucin production to protect against gastric inflammation and ulcers in mice [13,14].

Mitogen-activated protein kinases (MAPKs) and nuclear factor kappa B (NF-κB) are important signaling pathways involved in inflammation. Several studies have reported an enhanced expression and phosphorylation of MAPKs in the intestine of IBD patients [15,16,17]. Similarly, increased NF-κB expression in the intestinal epithelial cells is associated with inflammation in IBD patients [18]. In vitro and in vivo studies indicate that TQ can target kinase activity to suppress activation of MAPK pathways related to inflammation [19].

Previous studies have shown that TQ treatment mitigated colonic inflammation with a reduction in colonic myeloperoxidase activity, malondialdehyde, and increased glutathione levels in dextran sulfate sodium (DSS)-induced colitis in mice [20]. TQ treatment also reduced inflammation in acetic acid-induced colitis in rats, with inhibition of the myeloperoxidase activity, platelet-activating factor (PAF), and histamine, together with prevention of glutathione (GSH) depletion, suggesting that inhibition of oxidation was involved [21]. However, the underlying molecular mechanism of the anti-inflammatory effect mediated by TQ in colitis is still not clearly understood. Therefore, based on these considerations, we aimed to investigate the anti-inflammatory effect of TQ in colonic inflammation using an in vivo model and its effect was also evaluated in TNF-α-activated HT29 human colonic adenocarcinoma cells. The cancer cells were employed to confirm that TQ would also be an effective anti-inflammatory agent in human cells. We hypothesized that TQ alleviates colon inflammation by regulating the PPAR-γ and MAPK signaling pathways.

## 2. Materials and Methods

### 2.1. Chemicals and Reagents

Dextran sulfate sodium (DSS) (MW 36,000–50,000 kDa) was purchased from MP Biomedicals, Solon, OH, USA. Thymoquinone (cat# 274666-1G), hexadecyltrimethylammonium bromide (HTAB), and ortho-dianisidine dihydrochloride (ODD) were purchased from Sigma-Aldrich (St. Louis, MO, USA). IL-6 (cat# DY406), IL-1β (cat# DY401), and TNF-α (cat# DY410) ELISA kits purchased from R&D systems (Minneapolis, MN, USA). The reverse transcription kit was purchased from Applied Biosystems (Foster City, CA, USA). EvaGreen 5X Master Mix (cat# 08-36-00020) from Solis Bio-Dyne (Tartu, Estonia) and Macrogen Inc. (Seoul, South Korea), provided the master mix and primers for quantitative RT-PCR. The protease and phosphatase inhibitor were procured from Thermo-Scientific (Rockford, IL, USA). Antibodies (monoclonal; COX-2 (cat# sc-376861), iNOS (cat# sc-7271), ERK (cat# sc-514302), pERK (cat# sc-7383), JNK (cat# sc-7345), pJNK (cat# sc-293136), P38 (cat# sc-7972), pP38 (cat# sc-7973), NF-κB (cat# sc-8008), pNF-κB (cat# sc-136548), PPAR-γ (cat# sc-7273), and GAPDH (cat# sc-32233)) were purchased from Santacruz Biotechnology (Dallas, TX, USA). The Pierce BCA protein estimation kit, PVDF membrane, TRIZOL reagent, and West Pico super signal chemiluminescent substrate were purchased from Thermo-Scientific (Waltham, MA, USA). RIPA buffer was purchased from Millipore (St. Louis, MO, USA). Dulbecco’s modified Eagle’s medium was purchased from Pierce Hyclone (Fremont, CA, USA). Heat-inactivated fetal bovine serum was purchased from Gibco (Carlsbad, CA, USA). The Cell Titer-Glo^®^ Luminescent Cell Viability Assay Kit was purchased from Promega (Madison, WI, USA).

### 2.2. Animals

C57BL/6J mice (12 weeks old) weighing 25–30 g were supplied by the Central Animal Facility, CMHS, UAE University. Two animals per cage were housed for each experiment one week before the initiation of the study for acclimatization. The temperature was maintained at 23 ± 1 °C, a 12 h light–dark cycle, with 50–60% humidity. Food and water were provided *ad libitum*. The UAEU Institutional Review Board approved the studies (approval # ERA_2018_5742).

### 2.3. Experimental Design

Mice were randomly allocated to 5 groups with 8 animals in each group.

Group I: Untreated control. Group II: DSS alone. Group III: DSS + TQ (20 mg/kg body weight/day). Group IV: DSS + TQ (40 mg/kg body weight/day). Group V: DSS + SAZ (50 mg/kg body weight/day). DSS (2%) was prepared in autoclaved drinking water freshly every day. The treatment protocol was for 8 days. The disease activity index (DAI) was determined based on the score calculations provided in Table 1. At the end of the 8-day treatment protocol, the animals were euthanized using pentobarbital (100 mg/kg body weight). Colons were surgically removed, and the length measured and photographed, including the caecum. After removing the caecum, the colons were then cleaned with ice-cold saline to eliminate fecal content. After the cleaning, the colon was dissected to remove a 0.5 cm piece from the junction between the proximal and distal colon (the proximal colon has a mucosa with traverse folds until halfway and then continues as distal colon with longitudinal folds) [22]. These segments were fixed using 10% formalin for all histopathology studies. The remaining colon was subjected to mucosal scraping. Then, 25 mg of the freshly scraped colonic mucosa was used for an MPO assay as described below, and the rest of the mucosal scraping was snap-frozen immediately using liquid nitrogen and stored at −80 °C until further use.

### 2.4. Evaluation of Disease Activity Index (DAI) Score

Mice were weighed daily and observed for the presence of loose stools, diarrhea, and bleeding. The DAI scores obtained were based on parameters as published in our previous study [23]. In short, the DAI scores comprise the scores obtained from weight loss, diarrhea, and bleeding recorded daily as indicated in Table 1.

### 2.5. Proinflammatory Cytokines Estimation by Enzyme-Linked Immunosorbent Assay (ELISA)

Colonic mucosa was homogenized using RIPA buffer (cat# 20-188, Millipore, Burlington, Massachusetts, USA) with a phosphatase and protease inhibitor cocktail tablet (cat# A32959, Thermo-Scientific, USA), using zirconium beads (2 mm, cat# 11079124zx, BioSpec, Bartlesville, OK, USA) in a Precellys 24 tissue homogenizer (Bertin Instruments, Montigny-le-Bretonneux, France). The homogenate was then centrifuged at 1000 × *g* at 4 °C. The supernatants were transferred into a fresh microcentrifuge tube with gentle mixing on the tube rotator overnight at 4 °C. Then the homogenate was centrifuged at 15,000× *g* at 4 °C, and the supernatant was diluted (1:3) with RIPA buffer (cat# 20188, EMD Millipore, Billerica, MA, USA). The diluted homogenate protein concentration was determined using a Pierce BCA Protein Assay Kit (cat# 23225, Thermo-Scientific, Rockford, IL, USA). TNF-α, IL-1β, and IL-6 cytokines were measured in the obtained colonic mucosal homogenates by ELISA assay according to the manufacturer’s instructions.

### 2.6. Myeloperoxidase (MPO) Assay

Tissue MPO activity was performed as reported previously [24]. Briefly, 25 mg of each freshly scraped colonic mucosa was homogenized using zirconium beads in 50 mM phosphate buffer (pH6) containing 0.5% hexadecyltrimethylammonium bromide (HTAB). Homogenates went through a freeze–thaw cycle (liquid nitrogen and a 25 °C water bath) and were finally sonicated for 30 sec. This process was repeated three times. Then, suspensions were centrifuged at 20,000× *g* for 20 min at 4 °C. The supernatant (0.1 mL) was mixed in 2.9 mL of 50 mM phosphate buffer (pH 6) containing 0.53 mM of o-dianisidine hydrochloride and 0.15 mM hydrogen peroxide and the change in absorbance was measured every 15 s for 5 min at 460 nm. The results were expressed in units (U) of MPO/mg of protein. The protein concentrations of the supernatants were determined by bicinchoninic acid assay (Pierce, Rockford, IL, USA). MPO activity was calculated as mean absorbance at 460 nm/incubation time/protein concentration.

### 2.7. Histopathological Evaluation

A cleaned colon piece was fixed using 10% formaldehyde solution overnight. The dehydration process of the tissues was carried out using increasing concentrations of ethanol. The dehydrated tissues were embedded in paraffin. These paraffin embedded tissues were cut into thin slices (2 μm thick) and were stained using hematoxylin and eosin for the histological evaluation. To determine the histopathological changes, a clinical pathologist evaluated the scoring of each sample (Table 2). The samples were blinded for histopathological evaluation. The degree of colonic inflammation was scored using the method adapted from a previously published study [23].

### 2.8. RNA Extraction and Real-Time RT-PCR

Total RNA was extracted from the colon using the TRIZOL reagent, and cDNA conversion was carried out using the High-Capacity cDNA Reverse Transcription kit. Real-time polymerase chain reaction (PCR) was performed using the Quant Studio 7 Flex Real-Time PCR System (Thermo Fisher Scientific, MD, USA) with EvaGreen Master Mix [25]. The conditions for real-time PCR are 95 °C for 12 min, followed by 40 cycles of 95 °C for 15 s and 60 °C for 30 s and 72 °C for 30 s. The 18S transfer RNA was used as an internal reference gene. The CT values change was calculated using the delta CT method (2-ΔΔCT) [26]. Primer sequences for all genes used in the present study were reported in our previous study [25]. Primers for Human PPAR-γ (PMID 28986436) Forward: 5′-GGGCGATCTTGACAGGAAAG-3′, Reverse: 5′-CCCATCATTAAGGAATTCATGTCAT 3′; PPAR-γ (PMID20421464) Forward: 5′-TTGCTGAACGTGAAGCCCATCGAGG-3′, Reverse: 5′-GTCCTTAGATCTCCTGGAGCAG-3′.

### 2.9. Western Blot

Colonic mucosa (frozen) samples were homogenized using RIPA buffer (cat# 20188, EMD Millipore, Billerica, MA, USA) with a protease and phosphatase inhibitor cocktail (cat# A32961, Thermo-Scientific, Rockford, IL, USA) in a bead homogenizer, as previously described [25]. Then, 20 µg (colon tissue and HT-29 cells) proteins were resolved using sodium dodecyl sulfate-polyacrylamide gel electrophoresis (SDS-PAGE) using 8–12% gels and subsequently transferred onto PVDF membrane (cat# 88518, Thermo-Scientific, Rockford, IL, USA) using a Trans-Blot turbo transfer system (Bio-Rad, Hercules, CA, USA). Membranes were blocked with 5% BSA in Tris-buffered saline (TBS) containing 0.05% Tween-20 at 4 °C overnight. These PVDF membranes were immunoblotted using specific primary antibodies at 1:1000 (for all) dilution (p38, ERK, JNK, NF-κB (total and phosphorylated forms), COX-2, iNOS, PPAR-γ). This was followed by incubation with HRP-conjugated mouse IgG kappa binding protein (m-IgGκ BP- HRP: sc-516102) secondary antibody for 1 h at 1:10,000 concentration. The blots were developed using the Super Signal West Pico Plus chemiluminescent substrate (34577, Thermo-Scientific, Rockford, IL, USA). The blot images were acquired using a Sapphire Biomolecular Imager (Azure Biosystems, Dublin, CA, USA) using chemiluminescent detection of HRP. GAPDH (1:5000 dilution) was used as an internal control to normalize the blots.

### 2.10. HT-29 Cell Culture

The HT-29 cells were obtained from the American Type Culture Collection (Manassas, VA, USA). HT-29 cells were cultured at 37 °C and 5% CO_2_ in a humidified incubator in high-glucose DMEM, containing 100 U/mL penicillin, 100 µg/mL streptomycin, and 10% (*v/v*) heat-inactivated fetal bovine serum (GIBCO cat# 10500-064). These cultured HT-29 cells were seeded (1.5 × 10^5^ cells per well) onto 6-well plates 24 h before treatment. Cells were grown to 80% confluence. To induce inflammation, the HT-29 cells were treated with 1 ng/mL TNF-α with or without TQ (10 and 20 μM) for 24 h. The treated cells were collected for expression of inflammatory mediators (CXCL1, IL-8, and COX-2) and measurement of PPAR-γ mRNA and protein.

### 2.11. Cell Viability Assay

HT-29 cells (5000 cells/well) were seeded onto 96-well plates and treated with different TQ concentrations (0, 12.5, 50, 100, 150, and 200 µM) for 24 and 48 h. According to the manufacturer’s instructions, cell viability was determined using the Cell Titer-Glo kit after the treatment period. The luminescence was determined using the Tecan (Infinite 200 PRO) plate reader. The data are represented as percent of viable cells in the TQ treated groups compared to the untreated, control group.

### 2.12. PPAR-γ Promotor and Nanoluciferase Assay

Commercially available pNL1.3 and pNL1.3.CMV vectors were purchased from Promega (cat# N1021 and N1101, Madison, WI, USA) and the PPRE-pNL1.3 plasmid was purchased from Addgene (cat# 84394, Watertown, MA, USA). These vectors and plasmid (expressing luminescent NanoLuc^®^ luciferase) were transformed into JM109 competent bacterial cells by the ligation method and cultured overnight in 125 mL media at 37 °C. The plasmid was extracted using the Plasmid Purification Maxiprep kit (cat# 12162, Qiagen, Germany) following the manufacturer’s instructions. The plasmid DNA and Lipofectamine LTX mixture was prepared in serum and antibiotic-free DMEM media as described previously [27]. The transient transfection with PPRE-pNL1.3 or pNL1.3 basic secreted luciferase reporter used as control. HT-29 cells were transfected in 24-well plates (cat# 3516, Costar, Kennebunk, ME, USA) using Lipofectamine LTX Plus (Invitrogen, Carlsbad, CA, USA) following the manufacturer’s protocol at 37 °C. After 24 h, Lipofectamine LTX Plus added media were removed, replaced with fresh 2% FBS-media containing TQ 20 μM and 10 μM Rosiglitazone. After 24 and 48 h of treatment, 20 μL of each cell supernatant was dispensed into the wells of a black 96-well plate (cat# 237105, Thermo-Scientific, Roskilde, Denmark) and the secreted NanoLuc luciferase activity was determined using the Nano-Glo^®^ Luciferase assay buffer (cat# N1120, Promega, Madison, WI, USA) according to the manufacturer’s instructions. Luminescence in each well was then measured by using a Tecan Multimode plate reader.

### 2.13. Statistical Analysis

In vivo experiments: Mice were randomly allocated to 5 groups with 8 animals in each group, as described in the method section. The colon samples were collected from 8 different animals from each group. DAI determination, colon lengths were measured using 8 animals per group. After removal of the segment for histological examination (5 mm before to 5 mm after the junction between proximal and distal colon), the entire mucosal scraping from the rest of each colon was collected and pooled, *n* = 8 samples from each group. This mucosal tissue was used for MPO activity (measured immediately), and the rest snap frozen for subsequent extraction for ELISA assays, mRNA expression studies, and Western blotting (4 samples per group). Histological scoring was carried out using 3 thinly sliced (2.0 µM) sections from each colon, then the colon inflammation score was determined. *n* = 8 samples in each group were used for statistical analysis. In vitro experiments: HT-29 cells were grown in 6-well plates and challenged with TNF-α as described in the method section. The entire experiment was repeated 4 times, and *n* = 4 samples were collected for each experimental condition to determine statistical analysis. The statistical analysis was carried out using PRISM (Version 9) Software (GraphPad, San Diego, California, USA). The details concerning each distinct experimental design, number of samples, and experiment repeats are reported in the relevant method’s paragraphs and figure captions. One-way analysis of variance (ANOVA) for repeated measures was performed to determine the overall statistical differences among groups. Tukey’s post hoc test was used to determine the statistical significance of means of measures more stringently and, the results of these latter analyses, for each experimental group, are reported in the relevant graphs (* *p* <0.05, ** *p* < 0.01, *** *p* < 0.001, **** *p* <0.0001; *p* values 0.05 were accepted as statistically significant; NS: not significant; data are mean ± SEM).

## 3. Results

### 3.1. Effect of TQ on Disease Activity Index (DAI), Colon Length, and Myeloperoxidase (MPO) Activity

**Figure 1:**
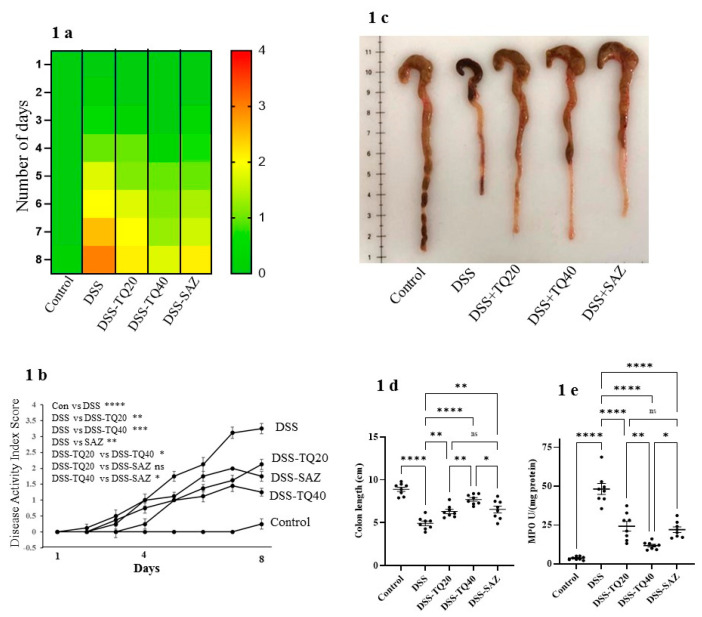
Effect of thymoquinone (TQ) on disease activity index (DAI), colonic length, and myeloperoxidase activity. (**a**) Shows the heat map of DAI; green color indicates no change in DAI scores, and red color indicates a severe increase in DAI scores. (**b**) DSS administration increased DAI scores significantly. TQ and sulfasalazine (SAZ) significantly limited the increase in DAI scores induced by DSS. (**c**,**d**) DSS markedly decreased the mean colon length. TQ and SAZ treatment partially prevented the decrease in colon length in the DSS group. (**e**) MPO activity was significantly higher in DSS groups. TQ and SAZ significantly decreased MPO activity in the DSS group. *n* = 8 animals used in each group to obtain the data. The data expressed as means ± SEM. **** *p* < 0.0001, *** *p* < 0.001, ** *p* < 0.01, and * *p* < 0.05. (NS) not significant.

The DSS-induced colitis group showed markedly increased DAI scores compared with the control group (*p* < 0.0001). TQ treatment (20 and 40 mg/kg body weight) in DSS-colitis animals dose-responsively (*p* < 0.01 and *p* < 0.0001) improved the DAI scores. As a positive control, SAZ treatment also showed a significant improvement in DAI score (Figure 1a,b) as shown in the heatmap and the line diagram.

The colonic length was markedly reduced in the DSS-administered group compared to control (*p* < 0.0001, Figure 1c,d). TQ (20 and 40 mg/kg bd wt) and SAZ markedly obtunded the shortening of colonic length (*p* < 0.0001, *p* < 0.01, Figure 1c,d) in the DSS-treated group. In the preliminary experiments, TQ treatment alone (40 mg/kg bd wt) had no effect on colonic length (data not shown).

The infiltration of neutrophils in the submucosa indicates the severity of inflammation. Secretion of the MPO enzyme is a commonly used marker from activated neutrophils to assess the inflammation in DSS-treated colon. DSS treatment markedly (*p* < 0.0001) increased MPO activity, indicating a high neutrophil infiltration level. TQ (20 and 40 mg/kg bd wt, Figure 1e) prevented the increase in MPO activity in a dose-dependent manner (TQ *p* < 0.0001, Figure 1e). SAZ treatment had a similar effect (SAZ *p* < 0.0001) but was significantly less potent than TQ (*p* < 0.05).

### 3.2. Effect of TQ on Microscopic Architecture of the Inflamed Colon

**Figure 2:**
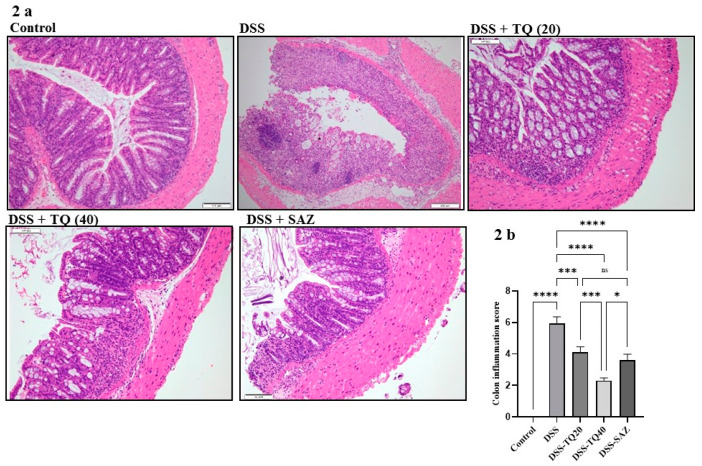
Effect of TQ on colon histology. (**a**) Histological examination shows typical colon architecture with normal thickness of submucosa, the muscle layer, and normal crypt and villi structure in control samples (scale bars: 100 µM). The DSS-administered colitis section shows loss of crypts and surface epithelium. The inflammation reaches the submucosa. TQ and SAZ treatment protected the microscopic architecture in the DSS-treated colitis group. (**b**) TQ treatment significantly reduced colon inflammation score. *n* = 8 animals used in each group to obtain the data. The data expressed as means ± SEM. **** *p* < 0.0001, *** *p* < 0.001, and * *p* < 0.05. (NS) not significant.

A well-formed colon mucosa villi and crypt architecture and normal thickness of the submucosa and muscle layer were observed in the healthy control group. In the DSS-treated group, colonic inflammation was observed in the submucosa with focal loss of crypt and the villi structures. The prominent histological observation in the DSS-treated group was marked crypt damage with submucosal edema and the widespread destruction of surface epithelium (Figure 2a). However, TQ treatment (20 and 40 mg/kg bd wt) and SAZ treatment showed minimal loss of crypt and villi epithelial architecture (Figure 2a,b). The colon inflammation score was significantly and dose-responsively lower in the TQ-treated groups (TQ *p* < 0.001, *p* < 0.0001). SAZ also reduced the colon inflammation score (*p* < 0.001), however, TQ was more potent (*p* < 0.05). The parameters used for the colon inflammation score are described in our previous study [25].

### 3.3. Effect of TQ on Proinflammatory Cytokines and Mediators

**Figure 3:**
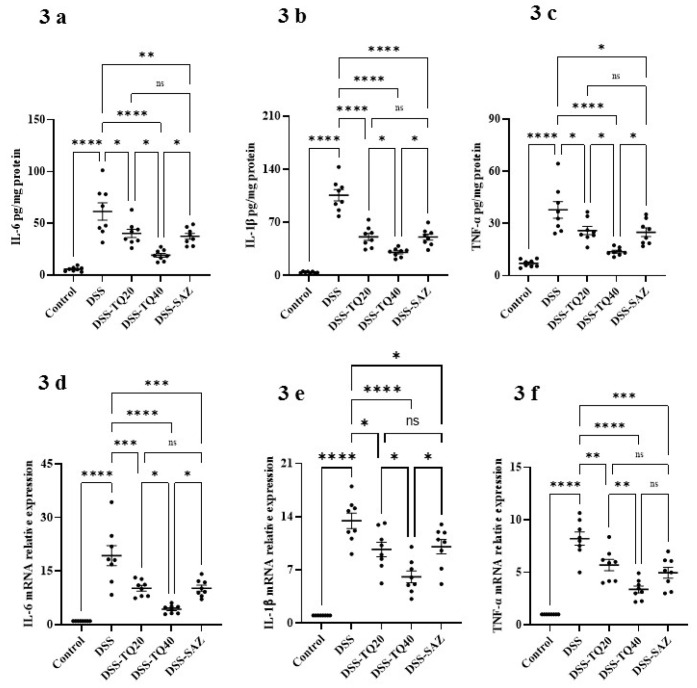

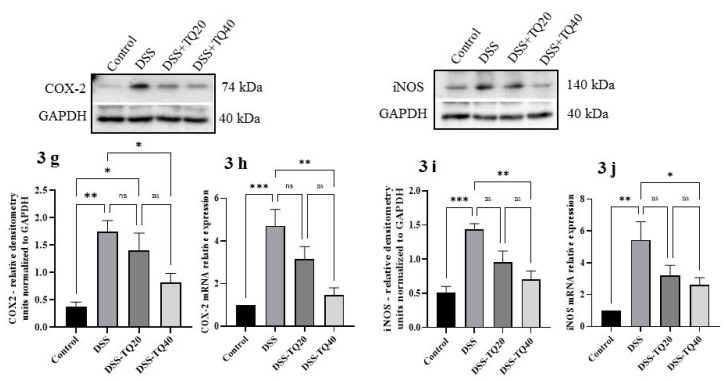
Effects of TQ on proinflammatory cytokines and mediator protein and mRNA expression. TQ treatment significantly prevented the DSS-induced increases in concentrations of proinflammatory cytokines both at protein (IL-6 (**a**), IL-1β (**b**), and TNF-α (**c**)) and mRNA expression levels (IL-6 (**d**), IL-1β (**e**), and TNF-α (**f**)) in DSS-induced colitis group. Similarly, TQ treatment also significantly decreased COX-2 and iNOS levels both at protein and mRNA levels in the DSS-induced colitis group (**g**–**j**). The density of the immunoblots was normalized to GAPDH as an internal control. *n* = 8 animals used for obtaining data for proinflammatory cytokines (IL-6, IL-1β, and TNF-α protein and mRNA) and *n* = 4 animals used in each group for proinflammatory mediators (COX-2 and iNOS protein and mRNA) expression studies. The data expressed as means ± SEM. **** p < 0.0001, *** p < 0.001, ** p < 0.01, and * *p* < 0.05. (NS) not significant.

DSS treatment significantly increased expression of the proinflammatory cytokines IL-6, IL-1β, and TNF-α (Figure 3a–c, *p* < 0.0001) compared with the control group. A concomitant increase in relative mRNA expression was observed for these respective cytokines (Figure 3d–f, *p* < 0.0001). TQ treatment (20 and 40 mg/kg bd wt) dose-responsively prevented the increases in these cytokine concentrations and their respective mRNA levels than the DSS-treated group. SAZ treatment also significantly reduced proinflammatory cytokine concentration. A similar decrease in respective cytokine mRNA expression was observed in SAZ treatment compared to the DSS-treated group. However, the higher dose of TQ was more effective at preventing the increase in production of all three cytokines, as well as the mRNA for IL-6 and IL-1β (*p* < 0.05).

The effects of TQ on DSS-treated animals was also evaluated on proinflammatory mediators such as COX-2 and iNOS at the protein (Figure 3g,i) and mRNA expression (Figure 3h,j) levels: COX-2 protein; *p* < 0.01; COX-2 mRNA; *p* < 0.001; iNOS protein; *p* < 0.001, iNOS mRNA; *p* < 0.01 were significantly increased in the DSS-administered group. TQ treatment significantly prevented the increase of COX-2 and iNOS protein expression at a higher dose (40 mg/kg bd wt) (COX-2 protein; *p* < 0.05; COX-2 mRNA; *p* < 0.01; iNOS protein; *p* < 0.01; iNOS mRNA; *p* < 0.05) in the DSS-treated group. However, a lower dose of TQ (20 mg/kg bd wt) did not have any significant effect in reducing the COX-2 and iNOS protein and mRNA expression.

### 3.4. Effect of TQ on MAPK Signaling Pathway and PPAR-γ Expression

**Figure 4:**
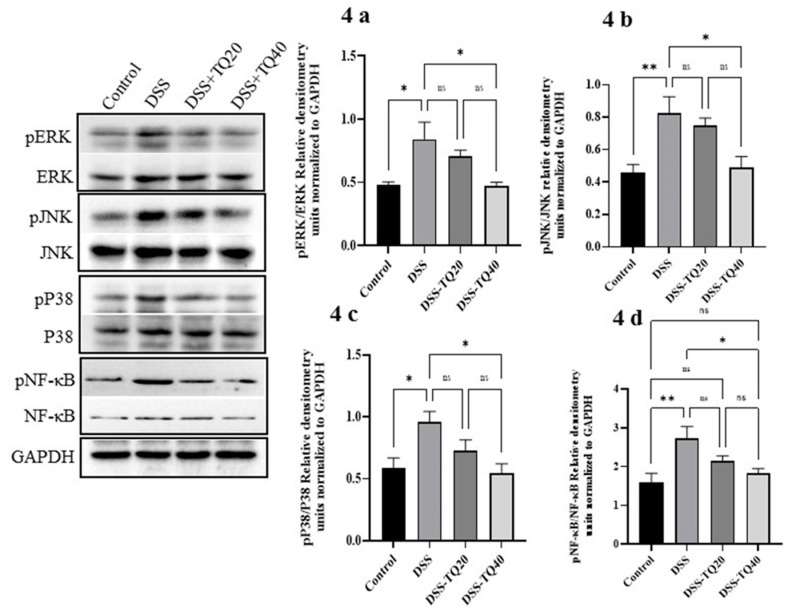

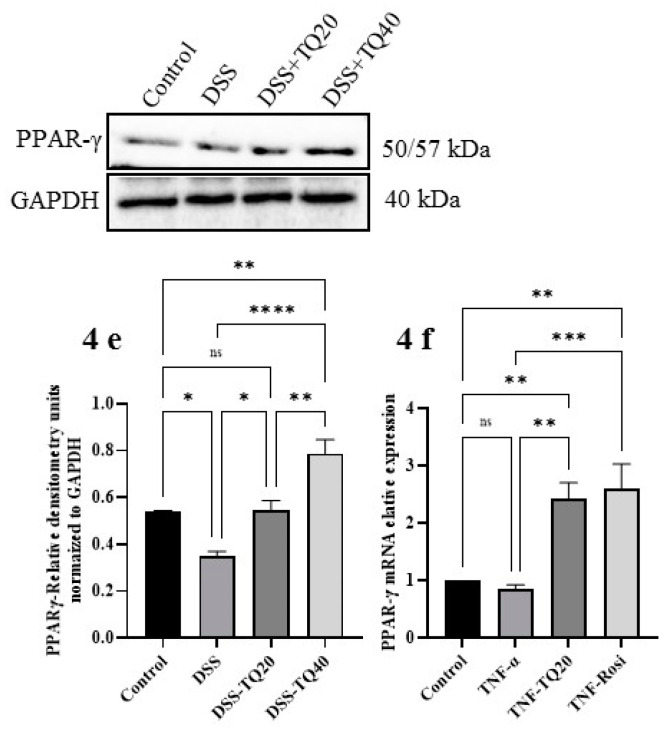
Effect of TQ on MAPK, NF-κB signaling pathways, and PPAR-γ protein and mRNA expression. (**a**–**c**) TQ treatment significantly prevented the increase in the phosphorylation of the protein kinase p-ERK, p-JNK, and p-p38 in the DSS-induced colitis group. Similarly, (**d**) TQ treatment significantly inhibited the increase in the phosphorylation of NF-κB protein in the DSS-induced colitis group. (**e**,**f**) TQ treatment significantly increased PPAR-γ expression both at protein and mRNA levels in the DSS-induced colitis group. The density of the immunoblots was normalized to GAPDH. *n* = 4 animals used in each group to obtain the data for protein and mRNA expression studies. The data expressed as means ± SEM. **** p < 0.0001, *** p < 0.001, ** p < 0.01, and * p < 0.05. (NS) not significant.

The role of the MAPK signaling pathway in DSS-treated colitis was determined by Western blot analysis of total ERK, JNK, and p38 protein expression, as well as their phosphorylated forms in the colon tissues (Figure 4a–c). A significant increase in the phosphorylated forms all three kinases (p-ERK, p-JNK, and p-p38) was observed in the DSS-treated group (*p* < 0.05 for p-ERK and p-p38; *p* < 0.01 for p-JNK). TQ treatment with a higher dose (40 mg/kg bd wt) showed a significant decrease in p-ERK, p-JNK, and p-p38 protein (*p* < 0.05 for all) in the DSS-treated group. The lower TQ treatment dose (20 mg/kg bd wt) failed to show a statistically significant decrease in the phosphorylation of these MAPK signaling pathway proteins. DSS treatment significantly (*p* < 0.01) increased phosphorylation of the NF-κB protein (Figure 4d; *p* < 0.05). The higher dose of TQ treatment (40 mg/kg bd wt) significantly decreased phospho-NF-κB protein (*p* < 0.05) compared to the DSS-treated group. However, the lower TQ treatment dose (20 mg/kg bd wt) failed to show a statistically significant decrease in phospho-NF-κB protein.

PPAR-γ is an important negative regulator of colon inflammation [1,2] and is highly expressed in the colonic epithelium [3]. Therefore, we investigated whether TQ modulates colonic epithelial PPAR-γ both at protein and mRNA expression levels (Figure 4e,f). DSS-treated colon showed a significant decrease in PPAR-γ expression (*p* < 0.05) compared to the control group. The TQ treatment significantly prevented the decrease of PPAR-γ protein and mRNA expression in the DSS-treated group (20 mg/kg bd wt; *p* < 0.01 protein, and *p* < 0.05 mRNA, 40 mg/kg bd wt; *p* < 0.01 for protein; *p* < 0.0001 for mRNA). These results indicate that the TQ positively regulated colon PPAR-γ expression both at protein and mRNA levels.

### 3.5. Effect of TQ on TNF-α Treated HT-29 Cells and PPAR-γ Expression

**Figure 5:**
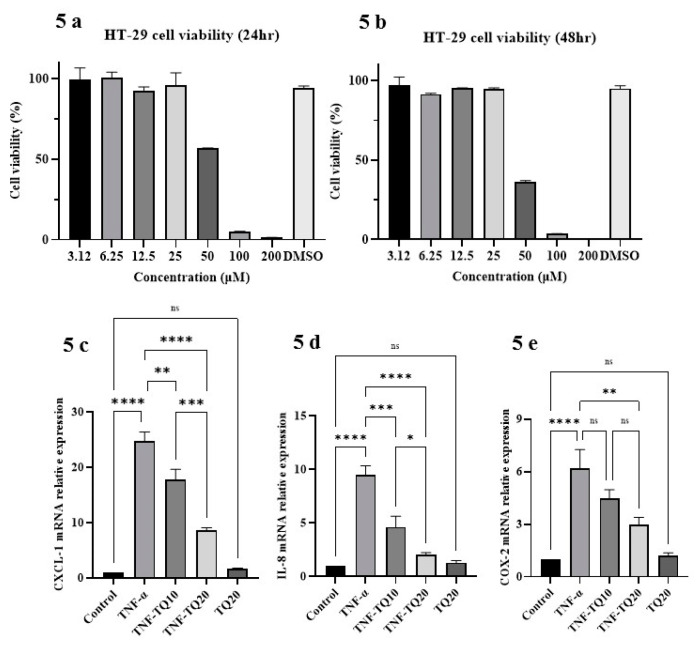

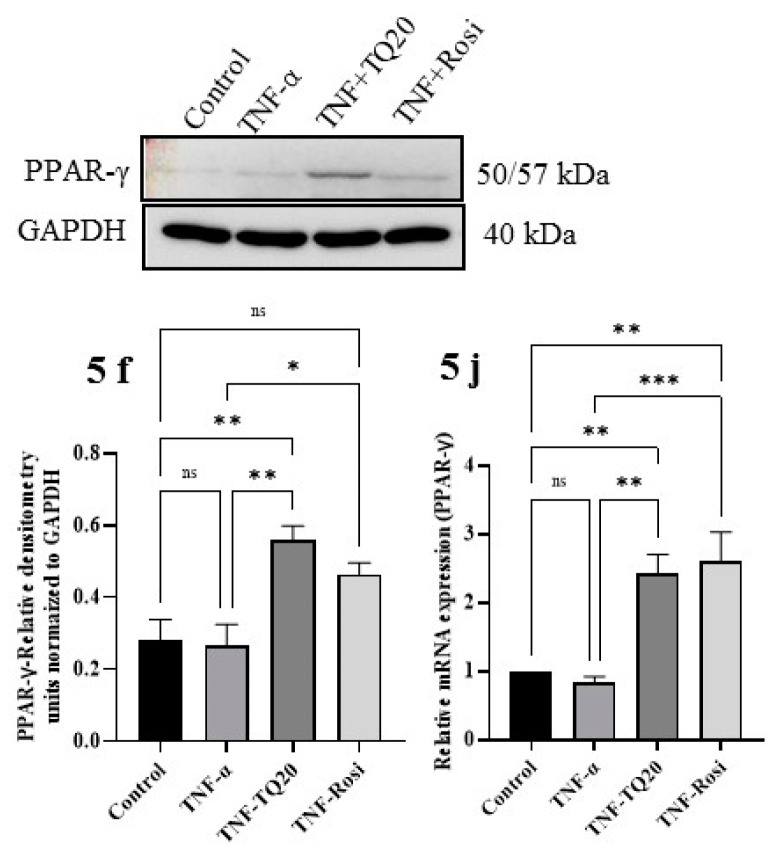
Effect of TQ on cell viability, proinflammatory chemokine mRNA, and PPAR-γ protein and mRNA expression in TNF-α challenged HT-29 human colon cancer cells. (**a**,**b**) A concentration-dependent cytotoxic effect of TQ was determined. (**c**–**e**) TQ treatment dose-dependently prevented the increase in CXCL-1, IL-8, chemokine, and COX-2 mRNA expression in TNF-α (1 ng/mL) challenged HT-29 cells. (**f**,**j**) TQ significantly increased PPAR-γ transcription factor protein, and mRNA expression in TNF-α challenged HT-29 cells. The density of the immunoblots was normalized to GAPDH. *n* = 4 separate experiments used for obtaining the data for protein and mRNA expression studies. The data expressed as means ± SEM. *p* ≤ 0.05 considered statistically significant. **** *p* < 0.0001, *** *p* < 0.001, ** *p* < 0.01, and * *p* < 0.05. (NS) not significant.

TNF-α stimulated HT-29 adenocarcinoma cells, a well-accepted in vitro model for human colon epithelial inflammation [4]. We investigated the effect of TQ on TNF-α treated inflammatory responses in this in vitro model. Therefore, to optimize cell model treatment protocol, initial experiments were undertaken to investigate TQ’s cytotoxic effect in HT-29 cells. The cellular monolayer was treated with different TQ concentrations (0 to 200 µM for 24 and 48 h). A significant cytotoxic effect was observed at 50 µM and above concentrations at both 24 and 48 h (Figure 5a,b). This is similar to results of a previous study [5]. Therefore, subsequent experiments were carried out using a non-cytotoxic range of 10 and 20 µM TQ concentrations. TNF-α-treated HT-29 cells exhibited a substantial increase in the mRNA of the proinflammatory genes such as CXCL-1, IL-8, and COX-2 (*p* < 0.0001 for all). The increased expression of the proinflammatory gene markers, CXCL-1, and IL-8 were significantly and concentration-dependently downregulated by TQ treatment (Figure 5c,d), whereas COX-2 mRNA expression was only downregulated by the higher dose of TQ (20 µM) (Figure 5e). In contrast, TQ (20 µM) alone did not influence any change in expression of these (CXCL-1, IL-8, and COX-2) proinflammatory chemokines in comparison to the control group. These results further confirm that TQ exerts a potent anti-inflammatory effect on human colonic cells when challenged with TNF-α similar to its effects in the mouse.

TQ’s effect on PPAR-γ was evaluated in HT-29 challenged with TNF-α. The higher TQ dose (20 µM) was used in subsequent experiments along with the known PPAR-γ stimulant, rosiglitazone (10 µM), as a positive control (Figure 5e,f). TQ significantly increased PPAR-γ expression both at protein and mRNA levels (*p* < 0.05 for protein; *p* < 0.01 for mRNA). Rosiglitazone caused a similar increase in PPAR-γ protein (*p* < 0.05) and mRNA (*p* < 0.01) expression.

### 3.6. Effect of TQ on PPAR-γ Promoter in HT-29 Cells

**Figure 6:**
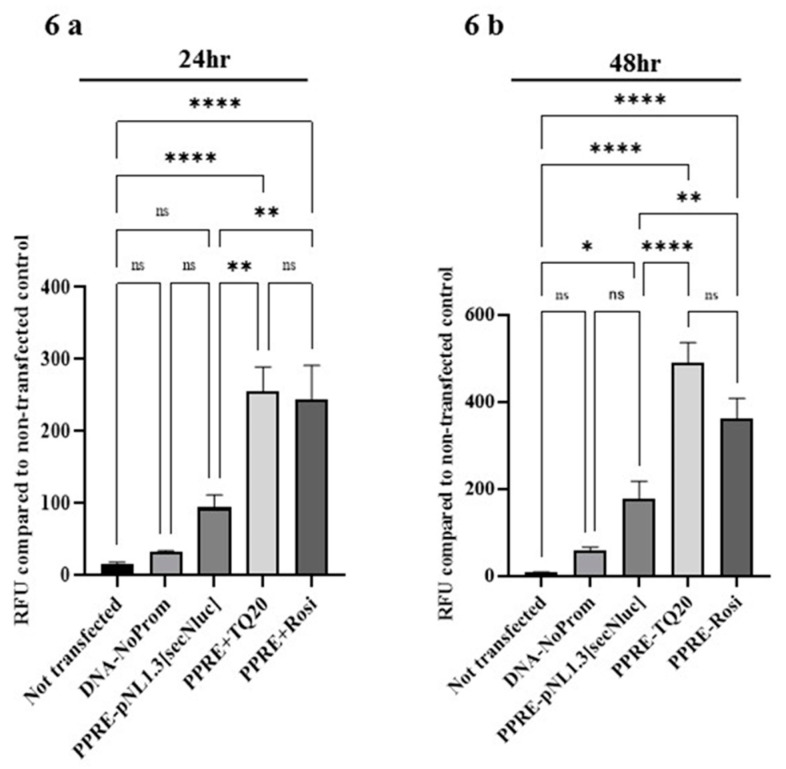
Effect of TQ on PPRE-pNL1.3[secNluc] plasmid containing PPAR-γ transcription factor promoter activity. The full-length sequence of PPAR-γ was cloned into PPRE-pNL1.3[secNluc] plasmid transfected using lipofectamine LTX in HT-29 cells. Luciferase expression at 24 and 48 h was determined in 50 µL supernatant culture media luminometrically. TQ treatment (20 µM) significantly increased luciferase expression in PPRE-pNL1.3 transfected HT-29 cells time-dependently (24 h (**a**), 48 h (**b**)). Rosiglitazone (PPAR-γ agonist, 10 µM) used as a positive control. The luciferase expression was compared to non-transfected controls. *n* = 4 separate experiments used for obtaining the data for PPAR-γ promoter activity studies. The data expressed as means ± SEM. ≤ 0.05 considered statistically significant. **** p < 0.0001, ** p < 0.01, and * p < 0.05. (NS) not significant.

TQ’s effect on PPAR-γ promoter was investigated to confirm whether the above-observed increase in PPAR-γ expression (both protein and mRNA level), was transcriptionally regulated. The PPAR-γ responsive element driving luciferase gene expression plasmid (PPRE-pNL1.3[secNluc]) was transfected into HT-29 cells. These transfected cells were treated with TQ (20 µM) and rosiglitazone (10 µM) for a 24 and 48 h period. The secreted luciferase luminescence was measured spectrophotometrically in 50 µl culture supernatant. Both TQ and rosiglitazone significantly (Figure 6a,b; *p* < 0.0001 for 24 and 48 h) increased luciferase luminescence intensity compared to non-transfected controls. These results indicate that TQ stimulates PPAR-γ promoter activity to drive increased PPAR-γ expression, suggesting its role in transcriptional regulation of this transcription factor.

## 4. Discussion

In the current study, we investigated the anti-inflammatory effects of TQ in in vivo and in vitro models of IBD and elucidated its molecular mechanisms of action. In vivo, TQ treatment improved DAI, preserved colon length and integrity, decreased MPO levels, and reduced the histopathological alterations, as indicated by minimal inflammatory cellular infiltration. These findings confirm TQ’s potent anti-inflammatory effects in this model [21].

The infiltration of macrophages in the colonic submucosa results in excessive secretion of proinflammatory cytokines such as TNF-α, IL-1β, and IL-6. These proinflammatory cytokines intensify colonic inflammation [28]. TNF-α production is mainly from monocyte/macrophage lineages and has proinflammatory and immunoregulatory properties [29]. Proinflammatory cytokines, such as IL-1β and TNF-α, are potent inducers of COX-2 expression, which produces proinflammatory eicosanoids [28]. TQ markedly prevented the increase in expression of these proinflammatory cytokines and COX-2. TQ treatment was previously shown to inhibit TNF-α and IL-6 production to limit inflammation in rheumatoid arthritis synovial fibroblasts [30]. The increase in tissue iNOS level observed in DSS-induced colitis was significantly reduced by TQ treatment. A similar reduction was also seen in LPS-activated RAW264.7 cells treated with TQ [31]. Collectively, these studies clearly indicate that TQ can mitigate the DSS-induced proinflammatory cytokine secretion in models of inflammation.

Many reports have indicated that mitogen-activated protein kinases (MAPK) act as regulators of production of inflammatory mediators in IBD models [32]. ERK^1/2^, c-Jun N-terminal kinase (JNK), and p38 belong to the MAPK family (serine/threonine protein kinases). Mitogens activate the ERKs, whereas JNK and p38 are activated by inflammatory stimuli and stress [33]. Inhibition of these inflammatory pathways is highly beneficial for colitis treatment. DSS-induced upregulated expression of the inflammatory cytokines TNF-α, IL-1β, and IL-6, the inflammatory mediators COX-2 and iNOS, as well as phosphorylation of ERK^1/2^, JNK, and p38. These were all significantly suppressed by TQ treatment. IL-1β has been shown to induce IL-6 secretion via MAPK signaling pathways [34,35]. The decreased IL-1β and IL-6 expression observed in the present study may be attributed to the downregulation of the MAPK signaling pathways. Previous studies using p38 and JNK inhibitors have demonstrated amelioration of DSS-induced colitis and inflammatory response [36,37,38]. These results indicate that the ability to inhibit activation of the MAPK pathways may at least in part explain the anti-inflammatory actions of TQ.

In addition to the regulatory effect of the MAPK signaling pathway, TQ also suppressed activation of NF-κB. NF-κB is a central controller of the inflammatory process by upregulation of diverse proinflammatory genes, such as TNF-α, IL-6, COX-2, and iNOS [39]. The NF-κB activation observed in the inflamed intestinal mucosa and the triggering of transcription of multiple proinflammatory mediators confirms its vital role in intestinal inflammation [40]. TQ has been reported to reduce IL-1β-induced inflammation in human osteoarthritis chondrocytes by suppressing the NF-κB and MAPK signaling pathways [41]. Therefore, the TQ inhibition of NF-κB activation is likely to have contributed to subduing the proinflammatory mediator response. The anti-inflammatory effects reported for rosiglitazone are thought mainly to be due to decreased expression of NF-κB and a concomitant increase in expression of the inhibitor, IκB. Presumably, the same is true of TQ.

As mentioned above, the peroxisome proliferator-activated receptors are highly expressed in the colon. PPAR-γ plays a vital role in regulating the inflammatory process by regulating the transcription of proinflammatory genes [42]. Decreased expression of PPAR-γ has been reported in colons of UC patients compared to healthy colons. Increased transcriptional activity of PPAR-γ is associated with decreased production of proinflammatory cytokines, such as IL-6 and IL-8, and chemokines, including CXCL1 (GRO-α), CXCL2 (MIP2-α), and CXCL3 (MIP-2β), in colonic tissues [6]. Our result indicates that TQ treatment increased PPAR-γ expression at protein and mRNA levels. TQ has been previously shown to upregulate PPAR-γ transcription factor in both in vivo and in vitro studies of a spinal cord injury (SCI) in a rat model [43], MCF-7 breast cancer cells [10], and T-cell acute lymphoblastic leukemia cells (Jurkat cells) [44].

The HT-29 adenocarcinoma cells, when differentiated, exhibit several cellular aspects of human colonic epithelial cells [45]. As an in vitro model of intestinal cells, HT-29 cells have some advantages and disadvantages. This cell line in its differentiated phenotype is similar to small intestine enterocytes with respect to brush border-associated hydrolases, while villin expression is close to the value observed in freshly prepared normal colonocytes. However, HT-29 cells cannot be compared entirely to normal enterocytes because they lack specific hydrolases such as lactase and maltase-glucoamylase [45]. Moreover, the expression of 377 genes in HT-29 cells compared with other intestinal cell lines that are also used as in vitro models of epithelium along with fresh tissue biopsies showed that differentiated HT-29 cells and human colonic tissue do not appear to be significantly different [46]. Many cellular receptors, such as opioids, serotonin, muscarinic, PPAR-γ, and PPAR-β/δ, are expressed in HT-29 cells. Our objective was to investigate the effect of TQ on the PPAR-γ transcription factor. Since it is highly expressed in HT-29 cells, as described by Tsukahara and Haniu [47], it becomes a valuable model system. Therefore, HT-29 cells challenged with TNF-α is a well-established colon epithelial inflammatory in vitro model for investigating the anti-inflammatory properties [48]. Cell toxicity assays indicated that TQ at higher concentrations induces significant cell death, and these results are in concurrence with a previously published study [49]. However, at a concentration (20 µM) with no cytotoxic effect, TQ suppressed CXCL-1, IL-8, and COX-2 mRNA expression in HT-29 cells exposed to TNF-α. Mucosal content of chemokines such as CXCL-1 and IL-8 are increased in IBD patients and are positively correlated with disease severity and tumorigenesis [50,51]. The chemokine IL8 (CXCL8), which can attract neutrophils to an inflammatory site, is secreted by HT-29 cells when challenged with TNF-α [52]. TQ has been previously shown to inhibit IL-8 and stimulate apoptosis of human hepatocellular carcinoma cell lines [53]. TQ also suppressed the production of CXCL1 in the lung cancer lines NCI-H460 and NCI-H146 [54].

The effect of TQ to increase PPAR-γ mRNA and protein expression was similar compared to that of the established PPAR-γ agonist, rosiglitazone. These findings indicate that TQ has potent PPAR-γ agonist activity. To investigate whether this observed agonist activity was due to transcriptional regulation, a PPAR-γ promoter assay was employed. The PPRE-pNL1.3 plasmid, a valuable tool for studying PPAR-γ activity in cell lines expressing PPAR-γ, was used in the present study [27]. TQ was shown to induce PPAR-γ promoter activity similarly to well-known PPAR-γ agonist, rosiglitazone. These results suggest that TQ could be considered as a potential ligand for PPAR-γ that reinforces the previous studies showing TQ-induced activity on PPAR-γ [9,10,43]. Anti-inflammatory effects combined with PPAR-γ agonist-like action may be a promising therapeutic approach to treat IBD. Therefore, modulating the expression of these receptors can become an attractive therapeutic target in IBD.

## 5. Conclusions

Our studies have confirmed the previous observations that TQ has potent anti-inflammatory effects in both in vitro and in vivo models of severe colonic inflammation. Furthermore, we have identified several molecular pathways involved in these anti-inflammatory effects, including upregulation and activation of PPAR-γ, suppression of MAP kinases, and downregulation of NF-κB. Thymoquinone or its analogues may be valuable drug candidates for the treatment of colitis.

## Figures and Tables

**Table 1:** Disease activity index score calculation.

Weight Loss	Score	Stool Consistency	Score	Rectal Bleeding	Score
No loss	0	Normal	0	No Blood	0
1–5%	1	Loose stool	2	Heme occult +ve and visual pellet bleeding	2
5–10%	2	Diarrhea	4	Gross bleeding and blood around anus	4
10–20%	3				
>20%	4				

**Table 2:** Colon tissue histological examination for inflammation.

Inflammation Graded	Percentage of Inflammation Involvement of Mucosal Surface Area		Hyperplastic Epithelium Graded Based on Extent of Involvement	
none	0	no inflammation	0	none
mild	1	1–25%	1	1–25%
moderate	2	26–50%	2	26–50%
severe	3	51–75%	3	51–75%
	4	76–100%	4	76–100%

## Data Availability

The data presented in this study are available on request from the corresponding author.
